# Activation of cannabinoid receptor 2 by turmeric oleoresin reduces inflammation and oxidative stress in an osteoarthritis *in vitro* model

**DOI:** 10.3389/fphar.2024.1488254

**Published:** 2024-12-09

**Authors:** Federico Ghiselli, Roberta Majer, Andrea Piva, Ester Grilli

**Affiliations:** ^1^ Vetagro S.P.A., Reggio Emilia, Italy; ^2^ Dipartimento di Scienze Mediche Veterinarie, Università di Bologna, Bologna, Italy; ^3^ Vetagro Inc., Chicago, IL, United States

**Keywords:** osteoarthritis, inflammation, turmeric, anti-inflammatory, antioxidant, endocannabinoid system, cannabinoid receptor 2

## Abstract

**Introduction:**

Osteoarthritis (OA) is a chronic degenerative joint disease characterized by the progressive degradation of articular cartilage, resulting in pain and reduced mobility. Turmeric (*Curcuma longa* L.) has been widely recognized for its anti-inflammatory and antioxidant properties, but the molecular mechanisms underlying its therapeutic effects remain inadequately explored. This study investigates the potential of turmeric oleoresin (TUR) to activate Cannabinoid Receptor 2 (CBR2) and its role in mediating anti-inflammatory and antioxidant effects in an *in vitro* OA model.

**Material and methods:**

Molecular docking and cAMP quantification assays were used to evaluate TUR’s agonistic activity on CBR2. Human chondrosarcoma cells (SW-1353) were treated with TUR under oxidative stress induced by menadione or inflammatory conditions simulated with IL-1β and TNF-α. The effects of TUR were assessed in the presence and absence of the CBR2 antagonist SR144528. Outcomes included changes in reactive oxygen species (ROS) production, inflammatory marker expression, oxidative defense markers and endocannabinoid system components and receptors.

**Results:**

TUR was confirmed as a CBR2 agonist and significantly reduced ROS production, downregulated pro-inflammatory cytokines (IL-6, COX-2, metalloproteases), and suppressed signaling pathways such as NFKB1, ERK 1/2, and c-Myc. These effects were reversed upon CBR2 inhibition. TUR also enhanced HMOX-1 expression and modulated endocannabinoid-related enzymes, highlighting its impact on oxidative stress and the endocannabinoid system.

**Discussion:**

These findings suggest that CBR2 activation is central to TUR’s anti-inflammatory and antioxidant effects. By modulating key pathways and endocannabinoid system components, TUR demonstrates potential as a novel therapeutic agent for OA management. Future studies could explore its clinical applications and further validate its molecular mechanisms *in vivo*.

## 1 Introduction

Osteoarthritis (OA) is a debilitating long-term condition that affects both older humans and animals. OA is characterized by the gradual erosion of articular cartilage, which is the smooth surface covering the ends of bones in joints, protecting them from friction and impact (Martel-Pelletier et al., 2016). This degeneration leads to restricted joint movement, severe pain, and ultimately disability. In humans, OA is widespread, affecting around 7% of the global population (Hunter et al., 2020; Primorac et al., 2021). The prevalence of OA in animals is even higher, estimated to be around 20% in dogs (Anderson et al., 2020) and over 90% in aged horses (30 years or older) (Ireland et al., 2012).

Despite the high prevalence of OA, the underlying mechanisms driving the disease are complex and multifactorial. OA often begins with trauma-caused microfractures or inflammation, leading to a slight increase in proteolytic activity in the cartilage. Molecules resulting from the breakdown of collagen and proteoglycans are absorbed by synovial macrophages and trigger the release of pro-inflammatory cytokines such as tumor necrosis factor-alpha (TNF-α), interleukin (IL)-1β, IL-6, and prostaglandins. These cytokines bind to chondrocyte receptors, triggering the release of matrix metalloproteases (MMP) and inhibiting type II collagen production, which accelerates cartilage degradation and leads to chondrocyte apoptosis. This disruption of homeostasis results in chronic inflammation that can cause pain and loss of mobility (He et al., 2020). Furthermore, OA involves the activation of several intracellular pathways, with studies identifying mitogen-activated protein kinase (MAPK) (Li et al., 2022), nuclear factor kappa-light-chain-enhancer of activated B cells (NF-κB) (Rigoglou and Papavassiliou, 2013), and extracellular signal-regulated kinases (ERK) 1/2 (Wang et al., 2011) as crucial mediators in the onset and progression of the disease. These pathways, along with others, amplify inflammatory responses and drive cartilage degradation and chondrocyte apoptosis, perpetuating the cycle of tissue damage and chronic inflammation in OA (Fazio et al., 2024).

OA is a challenging condition to treat, with no definitive cure discovered yet. Nonsteroidal anti-inflammatory drugs, pain management, and lifestyle modifications remain the primary therapeutic approaches for individuals with early-stage or mild-to-moderate OA. These measures aim to alleviate symptoms, improve joint function, and slow down the progression of the disease (Kolasinski et al., 2020).

Notably, molecules interacting with the endocannabinoid system (ECS) have garnered attention for their therapeutic potential in alleviating pain, mitigating oxidative stress, and reducing inflammation associated with OA and other bone-related conditions (La Porta et al., 2014; Gui et al., 2015; Xin et al., 2022). The ECS, a pervasive signaling network, plays a pivotal role in regulating pain perception, immune responses, inflammation, and various other essential bodily functions (Lu and Mackie, 2016).

Given the role of the ECS in regulating inflammation and pain, cannabinoid receptor (CBR) 2 has emerged as a crucial component in the context of OA (Turcotte et al., 2016). Predominantly expressed in immune cells, including synovial macrophages and chondrocytes, CBR2 activation has been shown to exert significant anti-inflammatory effects (Rzeczycki et al., 2021).

When activated, CBR2 can inhibit the production of proteins associated with inflammation such as IL-6, IL-8 (Capozzi et al., 2021), and MMP (Mariano et al., 2022). This anti-inflammatory action could be crucial to maintain joint health and mitigate the progression of OA. Recent research has increasingly focused on the therapeutic potential of targeting CBR2 to treat OA (Bryk and Starowicz, 2021). Studies have demonstrated that CBR2 agonists can significantly reduce pain and inflammation in animal models of OA, highlighting the receptor’s role in managing OA symptoms (Schuelert et al., 2010). These findings have led to the exploration of various CBR2 agonists as novel anti-inflammatory therapies (Bryk and Starowicz, 2021).

An intriguing alternative to synthetic molecules to activate the ECS involves the utilization of botanicals (Russo, 2016). Botanical compounds, including cannabinoids derived from *Cannabis spp.* and other natural sources, can exert their therapeutic effects by interacting with CBRs and modulating the intricate signaling pathways that regulate pain transmission and inflammatory processes (Russo, 2016; Almogi-Hazan and Or, 2020). Nevertheless, the therapeutic application of *Cannabis spp.* or its derived extracts is not always feasible due to legislative restrictions around the world (de Souza et al., 2022). In this context, the identification of natural compounds called “cannabinoid-like”, capable of interacting with the ECS and exerting anti-inflammatory and antioxidant actions, emerges as a viable alternative to cannabidiol and other compounds derived from *Cannabis spp.*

Turmeric, the yellow spice obtained from the root of the *Curcuma longa* L. plant, has long been valued for its medicinal properties, earning it a prominent position in traditional medicine practices (Hatcher et al., 2008). Turmeric has been central to traditional medicine systems like Ayurveda and Unani in India, South Asia, and Japan, has long been employed to alleviate symptoms of various inflammatory conditions, including OA (Bhowmik et al., 2009; Ansar et al., 2020). In Ayurvedic practice, turmeric is known as a “Rasayana” herb, which means it is used to promote overall health and longevity, and is specifically valued for its anti-inflammatory, antioxidant, and analgesic properties. In traditional Indian medicine, turmeric is consumed in various forms such as in milk or as a paste to treat biliary digestive disorder, wounds, relieve joint pain and improve mobility in patients suffering from arthritis (Chin, 2016).

Its most studied bioactive compound, curcumin (CUR - (1E,6E)-1,7-bis(4-hydroxy-3-methoxyphenyl)-1,6-heptadiene-3,5-dione), has gained significant scientific attention due to its remarkable anti-inflammatory and antioxidant effects (Hatcher et al., 2008). Several reviews have highlighted the positive effects of CUR against OA (Henrotin et al., 2013; Chin, 2016; Wu et al., 2019; Hsiao et al., 2021; Shokri-Mashhadi et al., 2021). The anti-inflammatory action of CUR arises from its multifaceted mode of action, targeting multiple molecular pathways involved in the inflammatory cascade (Peng et al., 2021). A key mechanism involves the inhibition of cyclooxygenase-2 (COX-2). By blocking COX-2 activity, CUR effectively reduces prostaglandin production, thereby dampening the inflammatory response (Rao, 2007). Alongside COX-2 inhibition, CUR exerts its anti-inflammatory effects by modulating the expression of pro-inflammatory cytokines, such as IL-1β, IL-6, and TNF-α, thereby limiting the inflammatory response (Gorabi et al., 2021). Moreover, recently Pawar et al. (2022) reported that CUR could selectively act on CBR2 as an agonist (Pawar et al., 2022). They reported that CUR treatment led to a decrease in inflammatory mediators, including IL-6, IL-1β, and TNF-α, within the myocardium of diabetic mice with myocardial infarction. This anti-inflammatory effect was inhibited by the CBR2 receptor antagonist AM630, suggesting that CUR’s anti-inflammatory action in the myocardium is mediated through the activation of the CBR2 receptor (Pawar et al., 2022).

Commercial turmeric extracts such as turmeric oleoresin also contain two important analogs of CUR: demethoxycurcumin [DMC - (1E,6E)-1-(4-hydroxy-3-methoxyphenyl)-7-(4-hydroxyphenyl)hepta-1,6-diene-3,5-dione] and bisdemethoxycurcumin [BDMC - (1E,6E)-1,7-bis(4-hydroxyphenyl)hepta-1,6-diene-3,5-dione] (Joshi et al., 2021). As extensively discussed in the review by Anand and colleagues (2008), numerous research teams have explored and compared the different beneficial properties of these compounds (Anand et al., 2008). In diverse contexts, CUR and its analogs have exhibited varying activities. For example, BDMC has shown stronger antitumor and antioxidant effects (Ruby et al., 1995) than CUR or DMC, as well as stronger activation of nuclear factor erythroid 2–related factor 2 (NRF2) mediated heme oxygenase −1 (HMOX-1) (Devasena et al., 2003) expression and inhibition of COX-dependent arachidonic acid metabolism (Hong et al., 2004).

Despite extensive research on the therapeutic potential of turmeric, there remains a significant gap in understanding its interaction with the ECS, particularly through CBR2, in the context of OA. The objective of this study was to investigate the ability of turmeric oleoresin to activate CBR2 and to understand the CBR2 role in modulating the anti-inflammatory and antioxidant properties of turmeric in an *in vitro* model of OA. The usage of a CBR2 antagonist aimed to determine whether inhibition of CBR2 alters the effects of turmeric, providing insights into the potential involvement of the ECS in turmeric’s beneficial properties against OA.

## 2 Materials and methods

### 2.1 Chemicals and reagents

Unless otherwise specified, chemicals and cell culture reagents were obtained from Merck Life Science S.r.l. (Milan, Italy).

Turmeric oleoresin (TUR) was purchased from Universal Oleoresins (Kerala, India). TUR contained 29.40% ± 4.20% (w/w) of total curcuminoids, of which 18.20% ± 2.60% (w/w) was curcumin (CUR), 6.01% ± 0.85% (w/w) was demethoxycurcumin (DMC), and 5.22% ± 0.74% (w/w) was bisdemethoxycurcumin (BDMC).

Stock solutions of β-Caryophyllene (BCP) and TUR were prepared in 100% ethanol at concentrations ensuring a final ethanol concentration of ≤0.5% (v/v) in the cell culture medium. Rimonabant, SR144528, 3-isobutyl-1-methylxanthine (IBMX), and forskolin (FSK) stock solutions were prepared in 100% dimethyl sulfoxide (DMSO) at a concentration that guarantees a final DMSO concentration ≤0.1% (v/v) in the cell culture medium. Vitamin C stock solution was prepared in water. All the solutions were stored at −20°C until use.

### 2.2 Cell line and culture conditions

The human chondrosarcoma cell line (SW-1353 – Cat. HTB-94™) was obtained from ATCC^®^ (American Type Culture Collection - Manassas, Virginia). SW-1353 cells were maintained at 37°C in an atmosphere containing 5% CO_2_ at 95% relative humidity. They were used between passages 20 and 30 to ensure consistent cell behavior. The basal medium was composed of Dulbecco’s Modified Eagle’s Medium high glucose, supplemented with 10% fetal bovine serum, 1% L-glutamine, 100 U/mL penicillin, 0.1 mg/mL streptomycin, and 1% non-essential amino acids. For all analyses, negative and positive control groups were incubated in a medium containing 0.5% (v/v) ethanol and/or 0.1% (v/v) DMSO, to exclude any possible solvent-mediated effect.

### 2.3 Viability assay

Cell viability was assessed using the PrestoBlue™ reagent (Thermo Fisher Scientific, Milan, Italy) according to the manufacturer’s guidelines. SW-1353 cells were seeded in 96-well plates at a density of 1 × 10^4^ cells/well and used 48 h post-seeding. They were then treated with TUR at increasing concentrations (n = 8) for 24 h, after which viability was assessed. Fluorescence values were recorded using a Varioskan™ LUX (Thermo Fisher Scientific, Milan, Italy). Cell viability was determined using the following formula:

$$Cell\;Viability\;\%=\frac{\text{Mean}\;\text{Treated}\;\text{Group}\;\text{Fluorescence}}{\text{Mean}\;\text{Negative}\;\text{Control}\;\text{Fluorescence}}\;x\;100$$

### 2.4 Cannabinoid receptors activation

#### 2.4.1 Cyclic adenosine monophosphate quantification

Intracellular cyclic adenosine monophosphate (cAMP) levels quantification was performed following the protocol described by Wang et al., 2004. Initially, cells were seeded into 24-well plates at a density of 5 × 10^4^ cells/well and used 48 h post-seeding. Rimonabant and SR144528 served as potent CBR1 (Porcu et al., 2018) and CBR2 (Rinaldi-Carmona et al., 1998) antagonists, respectively. The cells were divided into five groups (n = 6): (1) negative control, (2) positive control, (3) TUR, (4) TUR + Rimonabant, and (5) TUR + SR144528.

All groups were first treated with 100 μM of IBMX to inhibit the degradation of intracellular cAMP, together with 0.1 μM FSK for 10 min to induce cAMP production. Following this, the groups designated to receive the antagonists (groups 4 and 5) were treated with the respective antagonists at 1 μM for an additional 10 min. Finally, TUR at 15 ppm was added to groups 3, 4, and, 5, and cells were treated for another 30 min. After the challenge, cells were harvested, and cAMP was quantified using the cAMP Assay Kit (Competitive ELISA - Abcam plc., Cambridge, UK), following the manufacturer’s instructions. In this assay, BCP at 10 μM was used as a plant-derived CBR2 selective agonist control (Hashiesh et al., 2021), following the same protocol as described above.

#### 2.4.2 Molecular docking

Molecular docking was performed with CB-Dock2 online tool (https://cadd.labshare.cn/cb-dock2/index.php) (Liu et al., 2022; Yang et al., 2022) through the “Structure-based Blind Docking” function. The tool was used with default parameters, and the docking accuracy was validated using known CBR2 ligands from the study of Hua et al., 2020.

The CBR2 (PDB ID: 6KPF – Chain R) protein structure was downloaded from the PDB database (https://www.rcsb.org/) and the 3D molecular structures of Rimonabant, SR144528, BCP, CUR, DMC, and BDMC from PubChem (https://pubchem.ncbi.nlm.nih.gov/). The CBR2–ligand interactions were visualized using BIOVIA Discovery Studio Visualizer (https://discover.3ds.com/).

### 2.5 Oxidative stress challenge

Reactive oxygen species (ROS) levels were assessed using CellROX^®^ Deep Red Reagent (Thermo Fisher Scientific, Milan, Italy) following the manufacturer’s guidelines. Briefly, CellROX^®^ Deep Red Reagent is a cell-permeable, non-fluorescent reagent in its reduced state that becomes fluorescent upon oxidation by ROS, with an emission maximum of around 665 nm.

Cells were seeded into 96-well plates at a density of 1 × 10^4^ cells/well and subjected to a challenge 48 h post-seeding. The cells were categorized into four distinct groups (n = 8): (1) negative control, (2) positive control, (3) TUR, and (4) TUR + SR144528. The group exposed to the antagonist (group 4) underwent a pretreatment with SR144528 at 1 μM for 10 min. Then, both the TUR and TUR + SR144528 groups received treatment with TUR at 15 ppm for 2 h. Subsequently, menadione at 100 μM was introduced as a ROS-inducing molecule for 1 h in the presence of the treatment. Following this, the medium was replaced with fresh media containing CellROX^®^ Deep Red Reagent at 5 μM, and the cells were incubated for 30 min at 37 C. The medium was then removed, and the cells were washed three times with Dulbecco’s phosphate-buffered saline (DPBS). Fluorescence values were recorded using a Varioskan™ LUX (Thermo Fisher Scientific, Milan, Italy). Vitamin C at 150 μM served as a standard antioxidant internal control. Supplementary Figure S1 reports the oxidative stress challenge optimization.

### 2.6 Inflammatory challenge

Chondrocytes were challenged as described by Pang et al., 2021, with some modifications. Co-treatment with IL-1β and TNF-α was chosen to mimic the inflammatory environment of OA, as these cytokines are primary mediators of inflammation in OA and they activate a distinct set of OA-related genes (Shi et al., 2004).

Cells were seeded into 24-well plates at a density of 5 × 10^4^ cells/well and subjected to a challenge 48 h post-seeding. The cells were categorized into four distinct groups (n = 4): (1) negative control, (2) positive control, (3) TUR, and (4) TUR + SR144528. The group exposed to the antagonist (group 4) underwent a pretreatment with SR144528 at 1 μM for 10 min. Then, both the TUR and TUR + SR144528 groups received treatment with TUR at 15 ppm for 2 h. Following this, a challenge was induced using a mixture of IL-1β and TNF-α both at 10 ng/mL for 24 h, in the presence of treatments. After 24 h, cells were rinsed with DPBS, harvested for RNA extraction, and subjected to qPCR analysis. Supplementary Figure S2 reports the inflammatory challenge optimization.

### 2.7 qPCR

Total RNA extraction from challenged cells was performed using the NucleoSpin RNA kit (Macherey-Nagel Inc., Bethlehem, United States) following the manufacturer’s protocol. The RNA yield and quality were assessed spectrophotometrically by measuring absorbance at 260 and 280 nm with a Varioskan™ LUX (Thermo Fisher Scientific, Milan, Italy). Samples with a 260/280 ratio below 2.0 were excluded from subsequent analyses.

Afterward, the RNA underwent reverse transcription using the iScript cDNA synthesis kit (Bio-Rad Laboratories, Hercules, California, United States) according to the manufacturer’s instructions. For qPCR reactions, duplicate assays were conducted using the CFX Connect Real-Time PCR System and iTaq Universal SYBR Green Supermix (Bio-Rad Laboratories, Hercules, California, United States). Gene expression data were normalized using two reference genes: ribosomal protein L13 (RPL13) and TATA-binding protein (TBP). The 2^−ΔΔCT^ method (Livak and Schmittgen, 2001) was employed to calculate the gene expression fold change, which is reported in the results as a fold of change relative to the negative control group.

Details regarding primer sequences, expected product length, and GenBank accession numbers are provided in Table 1. Primers were designed using the Primer-BLAST tool (https://www.ncbi.nlm.nih.gov/tools/primer-blast/) and subsequently obtained from Merck Life Science S.r.l.

**TABLE 1:** Primers used for gene expression analysis. Primers were designed using the Primer-BLAST tool (https://www.ncbi.nlm.nih.gov/tools/primer-blast/) and subsequently obtained from Merck Life Science S.r.l. F, forward; R, reverse; CBR, Cannabinoid receptor; COX-2, Cyclooxygenase-2; DAGL-α, Diacylglycerol lipase alpha; HMOX-1, Heme oxygenase 1; IL, Interleukin; MAGL, Monoacylglycerol lipase; MMP, Metalloprotease; NFKB1, nuclear factor kappa B subunit 1; NRF2, Nuclear factor erythroid 2–related factor 2; PPAR-γ, peroxisome proliferator-activated receptor-γ; RPL13, Ribosomal protein L13; TBP, Tata binding protein.

Gene	Primer sequence (F and R) 5’ → 3′	Product length (bp)	Accession N
CBR1	F: CTGTTCCTCACAGCCATCGACA R: TGGCTATGGTCCACATCAGGCA	115	NM_016083.6
CBR2	F: AGTGTTGGCTGTGCTCCTCATC R: GTTGATGAGGCACAGCATGGAG	127	NM_001841.3
COX-2	F: TCCCTTGGGTGTCAAAGGTAAA R: TGGCCCTCGCTTATGATCTG	172	NM_000963.4
DAGL-α	F: AGAATGTCACCCTCGGAATGG R: GTGGCTCTCAGCTTGACAAAGG	115	NM_006133.3
HMOX-1	F: CCAGGCAGAGAATGCTGAGTTC R: AAGACTGGGCTCTCCTTGTTGC	144	NM_002133.3
IL-6	F: AGACAGCCACTCACCTCTTCAG R: TTCTGCCAGTGCCTCTTTGCTG	132	NM_000600.2
IL-8	F: GAGAGTGATTGAGAGTGGACCAC R: CACAACCCTCTGCACCCAGTTT	112	NM_000584.4
MAGL	F: ATGCAGAAAGACTACCCTGGGC R: TTATTCCGAGAGAGCACGC	245	NM_001,003,794.3
MMP1	F: ATGAAGCAGCCCAGATGTGGAG R: TGGTCCACATCTGCTCTTGGCA	137	NM_001145938.2
MMP13	F: CCTTGATGCCATTACCAGTCTCC R: AAACAGCTCCGCATCAACCTGC	97	NM_002427.4
MMP3	F: CACTCACAGACCTGACTCGGTT R: AAGCAGGATCACAGTTGGCTGG	156	NM_002422.5
NFKB1	F: CTGGAAGCACGAATGACAGA R: CCTTCTGCTTGCAAATAGGC	89	NM_001382627.1
NRF2	F: CACATCCAGTCAGAAACCAGTGG R: GGAATGTCTGCGCCAAAAGCTG	112	NM_006164.5
PPAR-γ	F: GATACACTGTCTGCAAACATATCACAA R: CCACGGAGCTGATCCCAA	91	NM_015869.4
RPL13	F: CTCAAGGTGTTTGACGGCATCC R: TACTTCCAGCCAACCTCGTGAG	143	NM_012423.4
TBP	F: TGTATCCACAGTGAATCTTGGTTG R: GGTTCGTGGCTCTCTTATCCTC	124	NM_003194.5

### 2.8 Immunofluorescence staining and quantification

Immunofluorescence (IF) staining was performed for total ERK, and cellular myelocytomatosis oncogene (c-Myc), using the protocol already reported by Ghiselli et al., 2021. Briefly, SW-1353 cells were seeded at a of 2.5 × 10^4^ cells/well onto Nunc Lab-Tek II Chamber Slide System (Thermo Fisher Scientific, Milan, Italy), and exposed to an inflammatory challenge as described in Section 2.6 *.* Cells were then fixed with 4% paraformaldehyde in DPBS for 20 min, followed by permeabilization using 0.5% Triton X-100 (VWR, Radnor, Pennsylvania, United States) for 15 min. Subsequently, the unspecific bond sites were blocked with 10% goat serum for 1 h. Primary monoclonal antibodies, as specified in Table 2, were diluted in a solution containing 2% bovine serum albumin and 0.05% saponins (Alfa Aesar, Haverhill, Massachusetts, United States) in DPBS. Cells were incubated with primary antibodies for 3 h at room temperature in a humidified chamber. Detection of bound primary antibodies was carried out using secondary antibodies conjugated to fluorescein isothiocyanate or tetramethylrhodamine for 1 h (dilutions reported in Table 2), followed by two washes with 0.2% bovine serum albumin and 0.05% saponins in DPBS. Finally, the slides were mounted with Fluoroshield containing 4′,6-diamidino-2′-phenylindole dihydrochloride (DAPI) to stain the nuclei. Images were captured from three different fields using a Nikon Eclipse Ci fluorescence upright microscope at either 20x or 40x magnification (Nikon Corporation - www.nikon.com) and processed using NIS-Elements software (Nikon Corporation - www.nikon.com). To quantify fluorescence intensity, images were analyzed using ImageJ2 (Rueden et al., 2017). For each group, the intensity of total ERK and c-Myc was measured by selecting four random square regions of interest (ROIs) in two different images. Background intensity was measured by selecting an area with no cells to ensure accurate correction. The integrated density for each ROI was calculated as:

Integrated density=Area of the selected ROI×ROI fluorescence

**TABLE 2:** Antibodies used for immunofluorescence assay. c-MYC, cellular myelocytomatosis oncogene; ERK, Extracellular signal-regulated kinases; FITC, Fluorescein isothiocyanate; TRITC, tetramethylrhodamine.

Antibody	Dilution	Supplier	Product number
Mouse anti-c-Myc	10 μg/mL	Thermo Fisher Scientific	MA1-16637
Rabbit anti-ERK1/2 (total ERK)	1 μg/mL	Thermo Fisher Scientific	MA5-15134
Goat anti-rabbit secondary antibody, FITC conjugated	4 μg/mL	Thermo Fisher Scientific	A27034
Donkey anti-mouse secondary antibody, TRITC conjugated	6 μg/mL	Thermo Fisher Scientific	A16016

The corrected total cell fluorescence (CTCF) was then determined using the following formula:

$$CTCF=Integrated\;density-\left(Area\;of\;the\;selected\;ROI\times Backgeound\;fluorescence\right)$$

### 2.9 Statistics and reproducibility

The figure legends provide detailed information regarding sample sizes and the statistical methods employed. All experiments were performed in duplicate to ensure reproducibility and accuracy of the results. All data are presented as mean ± standard error of the mean (SEM). Statistical analyses were performed using GraphPad Prism version 10.2.3. The distribution of data was assessed using the Shapiro-Wilk test, with *p*-values greater than 0.05 indicating a normal distribution. Outliers were identified and excluded using the ROUT method, with a False Discovery Rate (Q) set above 1%.

Each measurement was taken from distinct samples to ensure independent observations. In qPCR experiments, samples with a 260/280 ratio below 2.0 were excluded from the analysis to ensure RNA quality. The datasets for viability (n = 8), oxidative stress (n = 8), qPCR (n = 4), cAMP quantification (n = 6), and immunofluorescence quantification (n = 8) were normally distributed and analyzed using one-way ANOVA followed by Tukey’s multiple comparisons test. Statistical significance was set at *p* < 0.05.

## 3 Results

### 3.1 Optimal dosage selection

**FIGURE 1:**
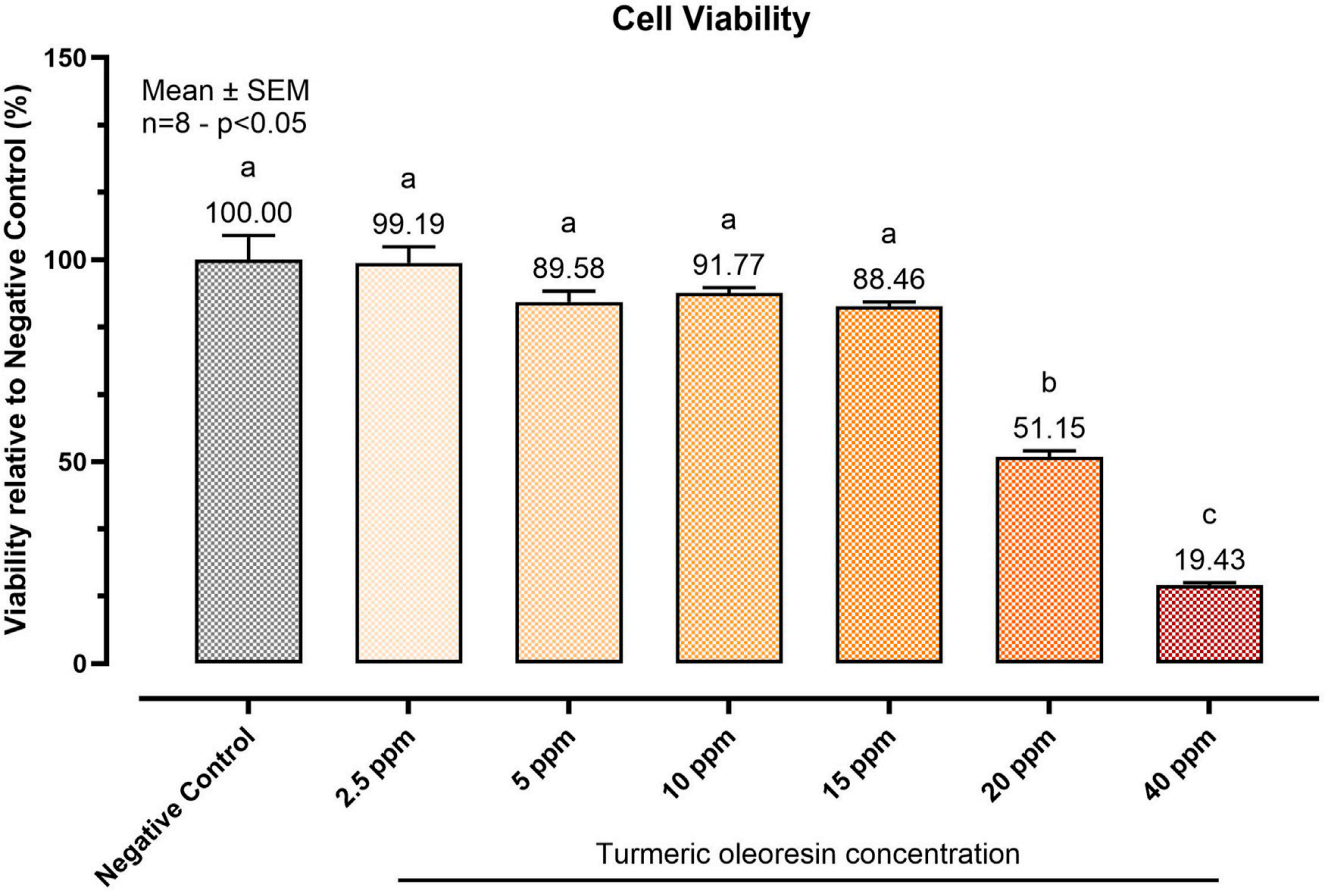
Effect of increasing concentrations of Turmeric Oleoresin (TUR) on SW-1353 cell viability. Cell viability was assessed using the PrestoBlue™ reagent after 24 h of treatment with varying TUR concentrations. Data are presented as mean ± SEM (n = 8) and as a percentage (%) relative to the negative control. Statistical significance was determined using one-way ANOVA followed by Tukey’s multiple comparisons test (*p* < 0.05). Different letters indicate significant differences.

To determine the highest safe dose of TUR for the study, a viability test was performed. Figure 1 reports the viability assay results. After 24 h of treatment, TUR concentrations above 15 ppm significantly reduced cell viability (*p* < 0.0001). Consequently, 15 ppm TUR was selected for subsequent analyses. This dose corresponded to 2.73 ± 0.39 ppm CUR (7.41 ± 1.06 μM), 0.90 ± 0.13 ppm DMC (2.66 ± 0.38 μM), and 0.79 ± 0.11 ppm BDMC (2.56 ± 0.36 μM).

### 3.2 Cannabinoid receptors’ activation by turmeric oleoresin

#### 3.2.1 cAMP production

The ability of TUR to interact with CBR1 and CBR2 was assessed by quantifying cyclic adenosine monophosphate (cAMP) levels, with BCP serving as an internal control.

CBR1 and CBR2 are G-coupled receptors that influence cellular signaling by modulating cAMP synthesis. Typically, CBR activation results in the inhibition of adenylyl cyclase, thereby decreasing forskolin-mediated cAMP production (Valenzano et al., 2005).

**FIGURE 2:**
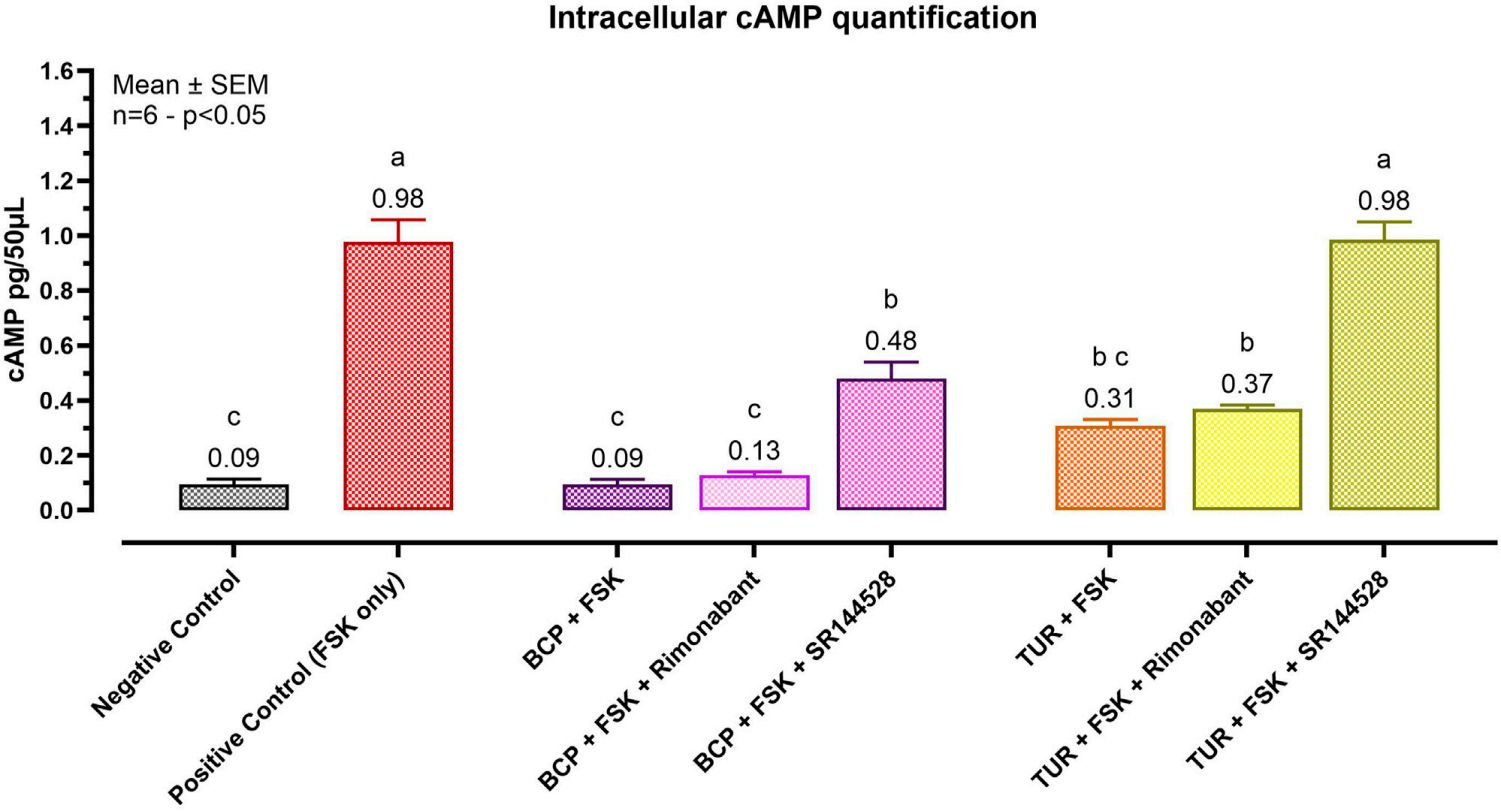
Effect of Turmeric Oleoresin on intracellular cAMP levels (pg/50 μL). All groups, except the negative control, were treated with 100 μM of 3-isobutyl-1-methylxanthine (IBMX) to inhibit cAMP degradation. Forskolin (FSK) at 0.1 μM was added for 10 min to induce cAMP production. Groups designated for antagonist treatment received 1 μM of either Rimonabant (CBR1 antagonist) or SR144528 (CBR2 antagonist) for 10 min before TUR (15 ppm) was added. Data are presented as mean ± SEM (n = 6) and analyzed using one-way ANOVA followed by Tukey’s multiple comparisons test (*p* < 0.05). Different letters indicate significant differences. BCP = β-caryophyllene; TUR = Turmeric oleoresin.

Figure 2 reports the cAMP assay results. In the positive control group, FSK induced a significant increase in cAMP levels (*p* < 0.0001) to 0.98 pg/50 μL. BCP, acting as a CBR2 agonist (internal control), maintained cAMP levels near those of the negative control. Notably, only pretreatment with SR144528 (a CBR2 selective antagonist) resulted in a significant (*p* = 0.0005) increase in intracellular cAMP levels, reaching 0.48 pg/50 μL (BCP + SR144528). Rimonabant showed no significant effects on cells treated with BCP, maintaining low cAMP levels (0.13 pg/50 μL).

TUR exhibited behavior similar to BCP, maintaining low levels of cAMP when treated with both FSK (0.31 pg/50 μL) and FSK + Rimonabant (0.37 pg/50 μL). Pretreatment with the CBR2 antagonist restored cAMP levels to those observed in the positive control (0.98 pg/50 μL).

#### 3.2.2 Molecular docking

**FIGURE 3:**
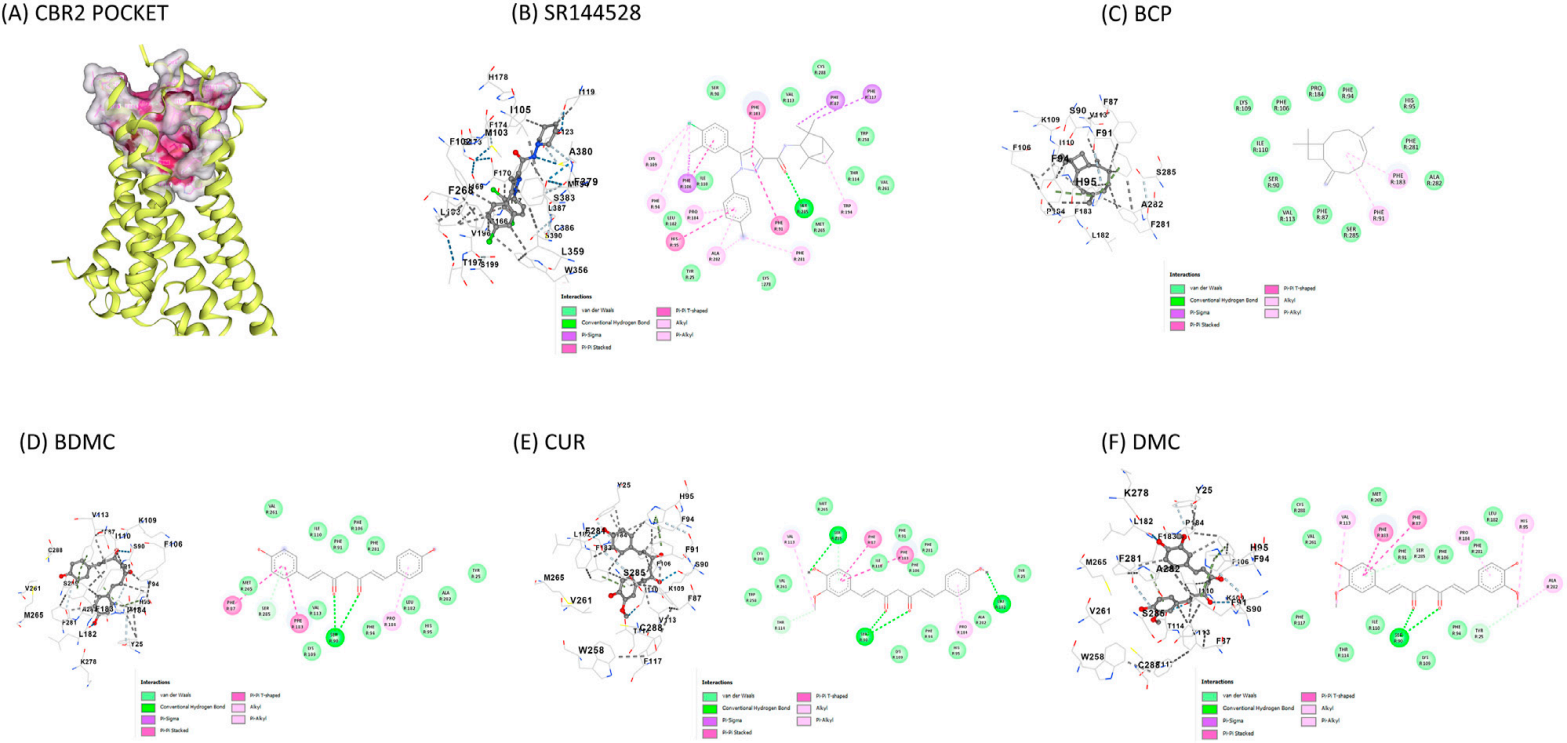
Visual representation of molecular docking on CBR2 using the CB-Dock2 tool **(A)** CBR2 binding pocket with highlighted important contact residues (PHE183, TRP256, PHE 281); **(B)** SR144528 binding to CBR2; **(C)** BCP binding to CBR2; **(D)** BDMC binding to CBR2; **(E)** CUR binding to CBR2; **(F)** DMC binding to CBR2. Binding energies and key contact residues are provided in Table 2. CBR2 = Cannabinoid-receptor 2; SR144528 = CBR2 antagonist; BDMC = Bisdemethoxycurcumin; BCP = β-caryophyllene; CUR = Curcumin; DMC = Demethoxycurcumin.

Table 3 and Figure 3 present the molecular docking results obtained using CB-Dock2 for various compounds with CBR2. The binding energies and key contact residues are summarized in Table 3. SR144528 exhibited the lowest predicted binding energy of −10.8 kcal/mol, and BCP recorded the highest binding energy of −9.1 kcal/mol. CUR and its analogs, BDMC and DMC, demonstrated binding energies of −9.6 to −9.7 kcal/mol.

**TABLE 3:** Binding energies and contact residues computed with CB-Dock2 on Cannabinoid receptor 2 (PDB ID: 6KPF). Important residues serving as “switches” for receptor activation are underlined. SR144528, CBR2 Antagonist.

Compound	Binding energy (kcal/mol)	Contact residues
SR144528	−10.8	TYR25, PHE87, SER90, PHE91, PHE94, HIS95, PHE106, LYS109, ILE110, VAL113, THR114, PHE117, LEU182, PHE183, PRO184, TRP194, TRP258, VAL261, MET265, LYS278, PHE281, ALA282, SER285, CYS288
Demethoxycurcumin	−9.7	TYR25, PHE87, SER90, PHE91, PHE94, HIS95, PHE106, LYS109, ILE110, VAL113, THR114, PHE117, LEU182, PHE183, PRO184, TRP258, VAL261, MET265, PHE281, SER285, CYS288
Bisdemethoxycurcumin	−9.7	TYR25, PHE87, SER90, PHE91, PHE94, HIS95, PHE106, LYS109, ILE110, VAL113, LEU182, PHE183, PRO184, TRP258, VAL261, MET265, LYS278, PHE281, ALA282, SER285, CYS288
Curcumin	−9.6	TYR25, PHE87, SER90, PHE91, PHE94, HIS95, PHE106, LYS109, ILE110, VAL113, THR114, PHE117, LEU182, PHE183, PRO184, TRP258, VAL261, MET265, LYS278, PHE281, ALA282, SER285, CYS288
β-caryophyllene	−9.1	PHE87, SER90, PHE91, PHE94, HIS95, PHE106, LYS109, ILE110, VAL113, LEU182, PHE183, PRO184, PHE281, ALA282, SER285

The primary contact residues identified by CB-Dock2 for each compound include several residues that are critical for CBR2 activation. Notably, TRP258 (Trp6.48), a crucial toggle switch residue for receptor activation, is consistently engaged by all compounds except BCP. Additionally, PHE183 (Phe5.47) and PHE281 (Phe7.35), which are important for stabilizing the active conformation, were identified as contact residues in all docking poses. Table 3 highlights these key residues.

Figure 3 represents the docking poses and interactions of CUR and its analogs on CBR2, along with the reference agonist BCP and antagonist SR144528. The binding pocket (cavity volume 2,273 Å^3^) and key contact residues are highlighted, showing significant interactions that align with the predicted binding energies. Supplementary Table S1 reports the molecular docking results on the CBR1 receptor.

### 3.3 Cannabinoid receptor 2 influence on turmeric bioactivity

#### 3.3.1 Antioxidant potential

**FIGURE 4:**
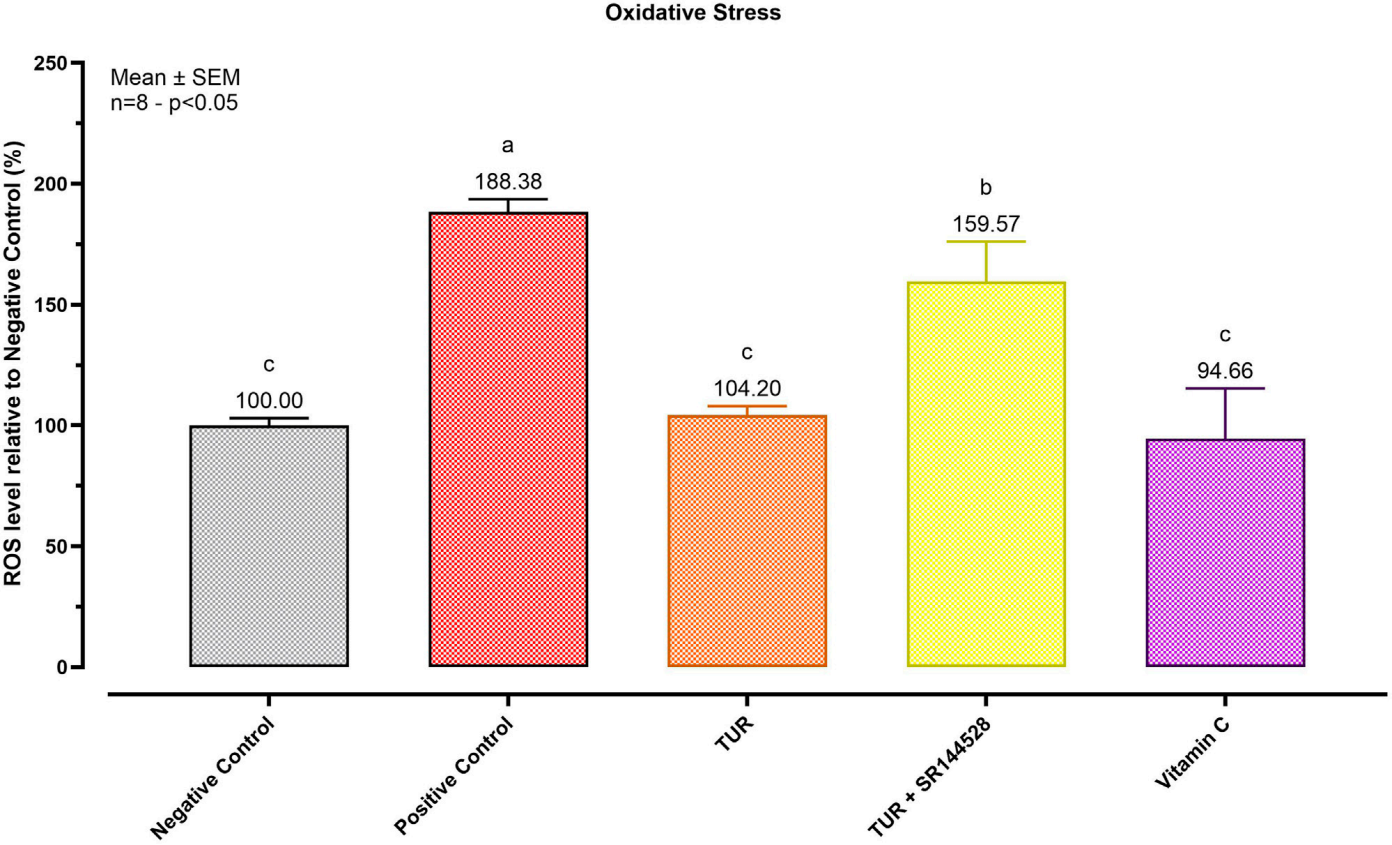
Antioxidant Potential of Turmeric Oleoresin (TUR). ROS levels were assessed using CellROX^®^ Deep Red Reagent. The group designated to receive the antagonist was pre-treated with 1 μM of SR144528 for 10 min. Then TUR (Turmeric oleoresin) at 15 ppm was added, and SW-1353 cells were incubated for 2 h before the challenge. Subsequently, menadione (100 μM) was added as a ROS-inducing agent for 1 h. Vitamin C (150 μM) was used as an internal antioxidant control. Data are presented as mean ± SEM (n = 8) and represent the ROS level percentage (%) relative to the negative control. Statistical analysis was performed using one-way ANOVA followed by Tukey’s multiple comparisons test (*p* < 0.05). Different letters indicate significant differences.

The antioxidant potential of TUR was evaluated by measuring intracellular ROS, as represented in Figure 4. Exposing SW-1353 cells to menadione for 1 h significantly increased intracellular ROS by 88.38% compared to the non-treated control (*p* < 0.0001). TUR significantly (*p* < 0.0001) blocked menadione-induced ROS generation, resulting in a non-significant increase of only 4.20% relative to the negative control. Pre-incubation with the CBR2 antagonist SR144528 reduced the antioxidant capabilities of TUR, leading to a 59.57% increase in ROS levels relative to the negative control (*p* < 0.0001) and 55.37% relative to the TUR-only group (*p* < 0.0001).

#### 3.3.2 Anti-inflammatory effect

**FIGURE 5:**
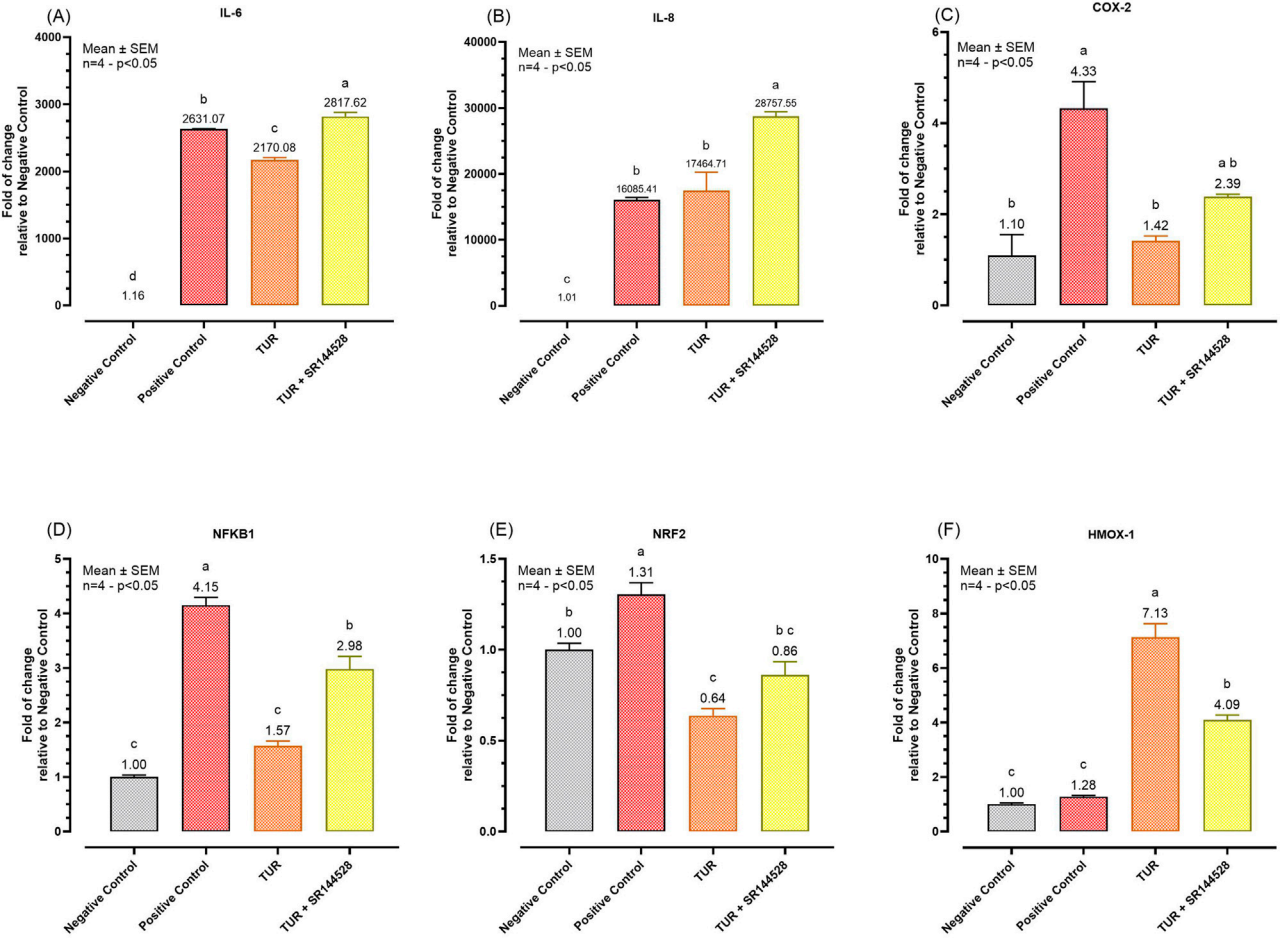
Gene expression of different inflammation markers. **(A)** IL-6 mRNA expression; **(B)** IL-8 mRNA expression; **(C)** COX-2 mRNA expression; **(D)** NFKB1 mRNA expression; **(E)** NRF2 mRNA expression; **(F)** HMOX-1 mRNA expression. The group designated to receive the antagonist was pre-treated with 1 μM of SR144528 for 10 min. Then TUR (Turmeric oleoresin) at 15 ppm was added, and SW-1353 cells were incubated for 2 h before the challenge. Subsequently, IL-1β and TNF-α both at 10 ng/mL (positive control) were introduced as an inflammatory stimulus for 24 h. Data are presented as mean ± SEM (n = 4) and represent the fold change relative to the negative control. Statistical analysis was performed using one-way ANOVA followed by Tukey’s multiple comparisons test (*p* < 0.05). Different letters indicate significant differences. IL = Interleukin; COX-2 = Cyclooxygenase-2; NRF2 = Nuclear factor erythroid 2-related factor 2; NFKB1 = Nuclear factor kappa B subunit 1; HMOX-1 = Heme oxygenase 1.

To assess the role of CBR2 activation in TUR’s anti-inflammatory action, gene expression analysis of key pro-inflammatory markers was conducted after a 24-hour inflammatory challenge. The results of the qPCR analysis are shown in Figure 5.

TUR treatment led to significant reductions in the expression of several pro-inflammatory markers compared to the challenged group (positive control). Specifically, TUR decreased IL-6 expression from 2631.07-fold in the positive control to 2170.08-fold (*p* < 0.0001, Figure 5A) which was also significantly different from the negative control (*p* < 0.0001, Figure 5A). However, TUR had no significant effect on IL-8, which remained elevated at 17464.71-fold, compared to the negative control (*p* < 0.0001, Figure 5B). In contrast, when TUR was combined with the CBR2 antagonist SR144528, IL-6 expression increased to 2817.62-fold (*p* < 0.0001, Figure 5A) and IL-8 expression rose further to 28757.55-fold (*p* = 0.0007, Figure 5B) compared to TUR alone, indicating a potential role of CBR2 in regulating these responses.

Moreover, TUR significantly reduced COX-2 expression from 4.33-fold in the positive control to 1.42-fold (*p* = 0.0035, Figure 5C), with no significant differences from the negative control in COX-2 expression. The combination of TUR and SR144528 resulted in a slight increase in COX-2 expression to 2.39-fold (Figure 5C) compared to TUR alone, suggesting that CBR2 activation contributes to TUR’s anti-inflammatory effects on COX-2.

TUR also significantly decreased nuclear factor kappa B subunit 1 (NFKB1) expression from 4.15-fold in the positive control to 1.57-fold (*p* < 0.0001, Figure 5D), reducing it to levels comparable with the negative control. When combined with SR144528, the reduction in NFKB1 expression was less pronounced, showing a 2.98-fold change (*p* = 0.0005, Figure 5D), further supporting the involvement of CBR2 in modulating NFKB1 expression.

Furthermore, TUR reduced NRF2 expression from 1.31-fold in the positive control to 0.64-fold (*p* < 0.0001, Figure 5E), which was also significantly lower than the negative control (*p* = 0.0025, Figure 5E). The presence of SR144528 moderated this reduction, resulting in a fold change of 0.86 (*p* < 0.0001, Figure 5E), suggesting a complex interaction between TUR and CBR2 in modulating NRF2 levels.

Finally, TUR significantly increased HMOX-1 expression from 1.28-fold in the positive control to 7.13-fold (*p* < 0.0001, Figure 5F). However, the presence of SR144528 reduced this effect to 4.09-fold (*p* < 0.0001, Figure 5F), reinforcing the importance of CBR2 activation in maximizing TUR’s potential.

**FIGURE 6:**
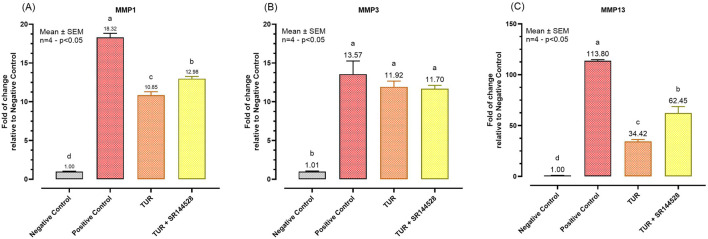
Gene expression of metalloproteases. **(A)** MMP1 mRNA expression; **(B)** MMP3 mRNA expression; **(C)** MMP3 mRNA expression. The group designated to receive the antagonist was pre-treated with 1 μM of SR144528 for 10 min. Then TUR (Turmeric oleoresin) at 15 ppm was added, and SW-1353 cells were incubated for 2 h before the challenge. Subsequently, IL-1β and TNF-α both at 10 ng/mL (positive control) were introduced as an inflammatory stimulus for 24 h. Data are presented as mean ± SEM (n = 4) and represent the fold change relative to the negative control. Statistical analysis was performed using one-way ANOVA followed by Tukey’s multiple comparisons test (*p* < 0.05). Different letters indicate significant differences. MMP = Matrix metalloprotease.

Figure 6 presents the qPCR results following the inflammatory challenge for the selected MMP. TUR treatment significantly lowered the expression of MMP1 and MMP13 compared to the positive control. Specifically, MMP1 expression decreased from 18.32-fold in the positive control to 10.85-fold with TUR treatment (*p* < 0.0001, Figure 6A), with levels significantly different from the negative control (*p* < 0.0001, Figure 6A). Similarly, MMP13 expression was drastically reduced from 113.80-fold in the positive control to 34.42-fold with TUR treatment (*p* < 0.0001, Figure 6C), also significantly different from the negative control (*p* < 0.0001, Figure 6C). However, pretreatment with the CBR2 antagonist SR144528 partially reversed these effects, increasing MMP1 expression to 12.98-fold (*p* = 0.0034, Figure 6A) and MMP13 expression to 62.45-fold (*p* = 0.0003, Figure 6C) compared to TUR alone, suggesting that CBR2 activation plays a role in TUR’s ability to suppress these MMP.

No significant changes were observed in MMP3 expression across the different treatment groups, indicating that TUR and CBR2 activation do not significantly influence MMP3 under the conditions tested (Figure 6B).

#### 3.3.3 Effects on ECS enzymes and receptors

**FIGURE 7:**
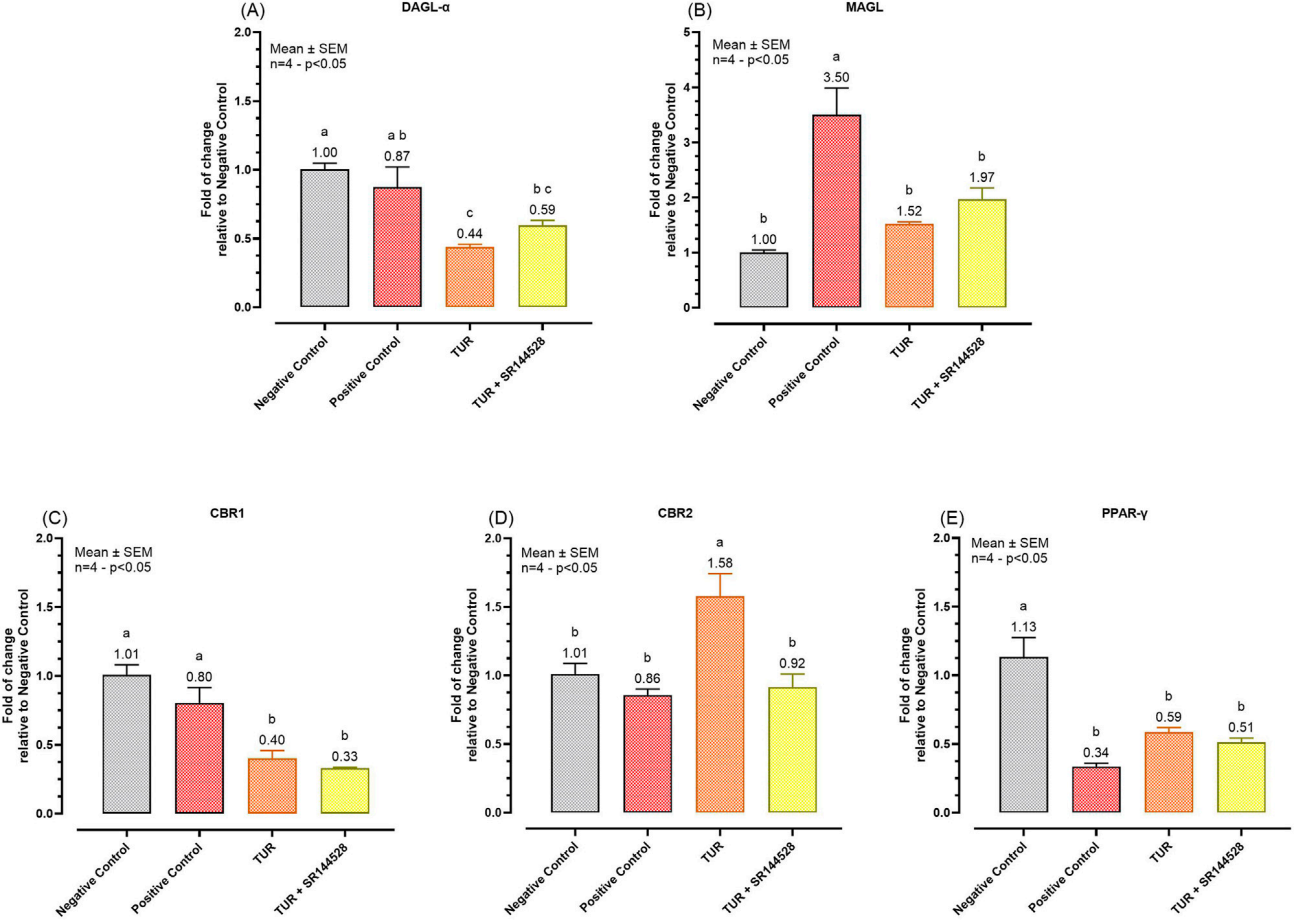
Gene expression of selected ECS enzymes and receptors. **(A)** DAGL-α mRNA expression; **(B)** MAGL mRNA expression; **(C)** CBR1 mRNA expression; **(D)** CBR2 mRNA expression; **(E)** PPAR-γ mRNA expression. The group designated to receive the antagonist was pre-treated with 1 μM of SR144528 for 10 min. Then TUR (Turmeric oleoresin) at 15 ppm was added, and SW-1353 cells were incubated for 2 h before the challenge. Subsequently, IL-1β and TNF-α both at 10 ng/mL (positive control) were introduced as an inflammatory stimulus for 24 h. Data are presented as mean ± SEM (*n* = 4) and represent the fold change relative to the negative control. Statistical analysis was performed using one-way ANOVA followed by Tukey’s multiple comparisons test (*p* < 0.05). Different letters indicate significant differences. DAGL-α = Diacylglycerol lipase-alpha; MAGL = Monoacylglycerol lipase; CBR = Cannabinoid receptor; PPAR-γ = Peroxisome proliferator-activated receptor-γ.

Figure 7 presents the qPCR results following the inflammatory challenge for the selected ECS enzymes and receptors.

TUR treatment significantly influenced the expression of various ECS enzymes and receptors. Specifically, TUR decreased DAGL-α expression from 0.87-fold in the positive control to 0.44-fold (*p* = 0.0112, Figure 7A). DAGL-α expression levels of the TUR treated group were also significantly lower compared to the negative control group (*p* = 0.0016, Figure 7A). However, when SR144528 was added, the decrease in DAGL-α expression was less pronounced, resulting in a non-significant fold change of 0.59 (Figure 7A) compared to TUR alone. For MAGL, TUR treatment reduced its expression from 3.50-fold in the positive control to 1.52-fold (*p* = 0.0051, Figure 7B), near to the negative control levels. The presence of SR144528 produced a non-significant increase in MAGL expression, showing a fold change of 1.97 compared to TUR alone (Figure 7B).

Regarding cannabinoid receptor mRNA expression, TUR significantly reduced CBR1 expression from 0.80-fold in the positive control to 0.40-fold (*p* = 0.0126, Figure 7C). Conversely, TUR increased CBR2 expression from 0.86-fold in the positive control to 1.58-fold (*p* = 0.0098, Figure 7D). The addition of the CBR2 antagonist SR144528 reversed this upregulation, bringing CBR2 expression back near the positive control level with a fold change of 0.92 (*p* < 0.0001, Figure 7D).

Lastly, no significant effects were observed on PPAR-γ expression across the different treatments, indicating that TUR and CBR2 activation do not significantly impact PPAR-γ expression under these experimental conditions (Figure 7E).

#### 3.3.4 Total ERK and c-MYC modulation

**FIGURE 8:**
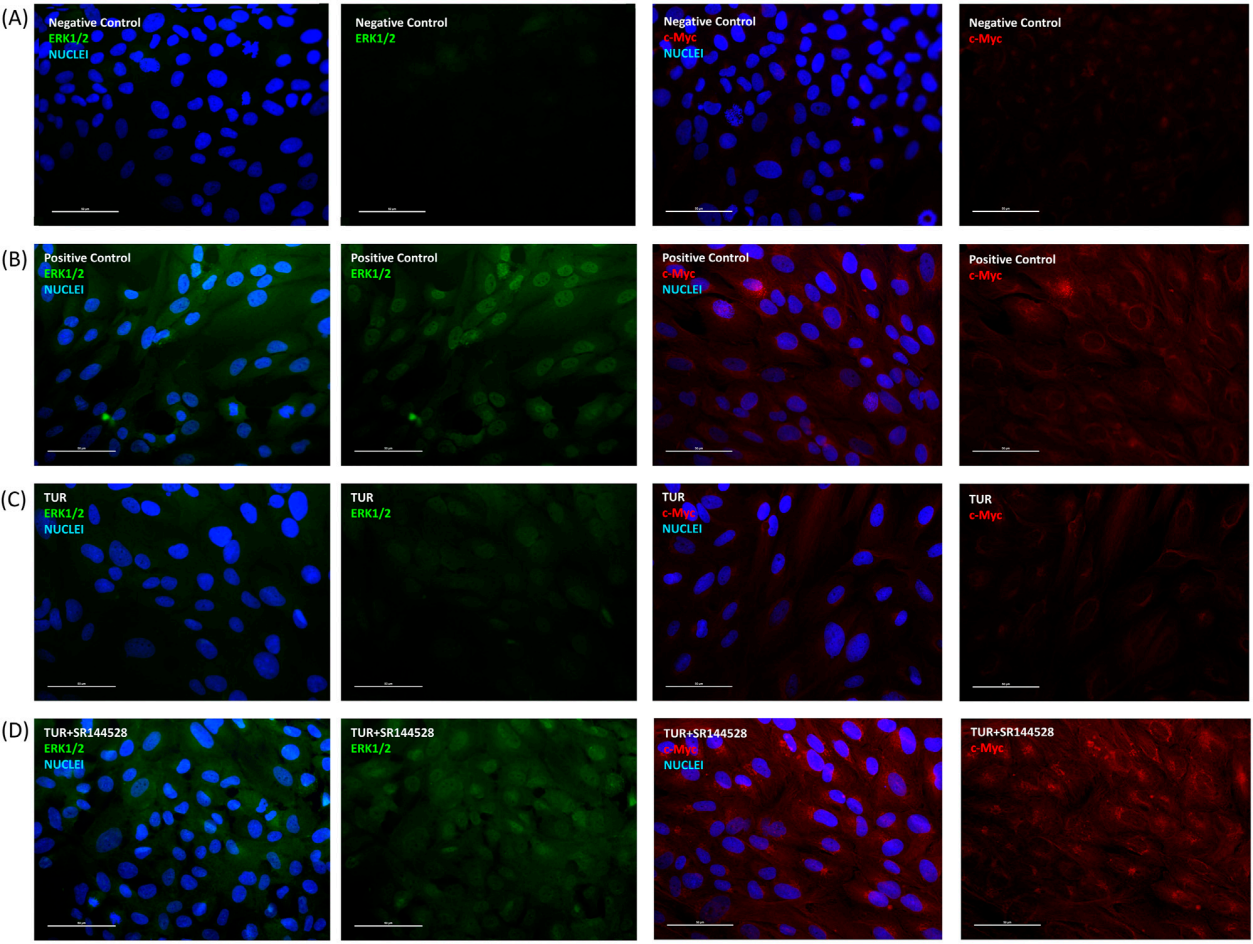
Immunofluorescence staining after inflammatory challenge. The group designated to receive the antagonist was pre-treated with 1 μM of SR144528 for 10 min. Then TUR (Turmeric oleoresin) at 15 ppm was added, and SW-1353 cells were incubated for 2 h before the challenge. Subsequently, IL-1β and TNF-α both at 10 ng/mL (positive control) were introduced as an inflammatory stimulus for 24 h. Images show nuclei (blue), total ERK (green), and c-Myc (red) in **(A)** negative control, **(B)** positive control, **(C)** TUR, and **(D)** TUR + SR144528 treated cells. Images were taken at ×40 magnification in three different fields. The scale bar corresponds to 50 μm. c-Myc = Cellular myelocytomatosis oncogene; ERK = Extracellular signal-regulated kinases.

Figure 8 illustrates the immunofluorescence of total ERK and c-MYC proteins following an inflammatory challenge, highlighting the effect of TUR treatment and CB2 receptor involvement. In the positive control (Figure 8B), the inflammatory challenge significantly increased the expression of both total ERK and c-MYC, confirming the induction of an inflammatory response. Treatment with TUR (Figure 8C) markedly reduced ERK and c-MYC levels, indicating TUR’s strong anti-inflammatory action. However, pretreatment with the CB2 receptor antagonist SR144528 (Figure 8D) prevented this reduction, maintaining elevated expression of both markers.

**FIGURE 9:**
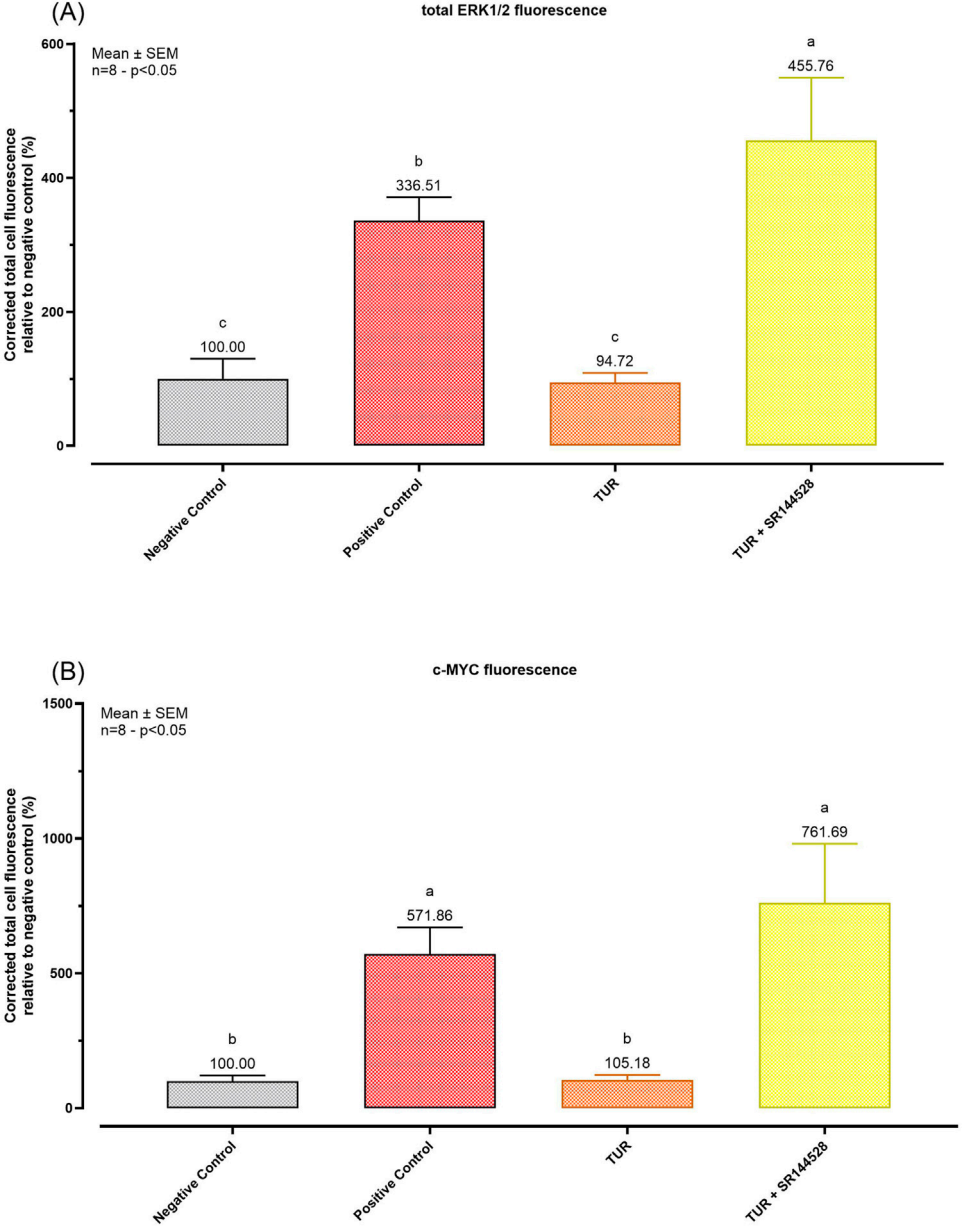
Immunofluorescence staining quantification. **(A)** total ERK1/2 fluorescence quantification; **(B)** c-MYC fluorescence quantification. The group designated to receive the antagonist was pre-treated with 1 μM of SR144528 for 10 min. Then TUR (Turmeric oleoresin) at 15 ppm was added, and SW-1353 cells were incubated for 2 h before the challenge. Subsequently, IL-1β and TNF-α both at 10 ng/mL (positive control) were introduced as an inflammatory stimulus for 24 h. Data are presented as mean ± SEM (*n* = 8) and represent the corrected total cell fluorescence (CTCF) percentage (%) relative to negative control. Statistical analysis was performed using one-way ANOVA followed by Tukey’s multiple comparisons test (*p* < 0.05). c-Myc = Cellular myelocytomatosis oncogene; ERK = Extracellular signal-regulated kinases.

Quantitative analysis shown in Figure 9 confirms these observations. TUR treatment reduced total ERK expression from 336.51% in the positive control to 94.72% (*p* < 0.0001, Figure 9A), aligning with levels observed in the negative control. In contrast, when TUR was combined with SR144528, total ERK expression increased significantly, reaching 455.76% relative to the negative control (*p* < 0.0001, Figure 9A). Similarly, TUR reduced c-MYC expression from 571.86% in the positive control to 105.18% (*p* < 0.0001, Figure 9B), comparable to negative control levels. With SR144528, c-MYC expression rose to 761.69% relative to the negative control (*p* < 0.0001, Figure 9B), nullifying TUR’s effect.

These results suggest that TUR’s effects on ERK and c-MYC are mediated through CBR2 receptor activation, as inhibition of CBR2 reverses TUR’s impact on these pro-inflammatory markers.

## 4 Discussion

### 4.1 Rationale

CUR, the main bioactive compound of turmeric, has gained significant scientific consideration for its remarkable anti-inflammatory and antioxidant effects, thus helping to slow down OA progression (Russo, 2016). Turmeric extracts such as TUR contain CUR and two important analogs of CUR: BDMC and DMC, which have exhibited varying interesting activities (Almogi-Hazan and Or, 2020). Some research groups have published systematic reviews and meta-analyses comparing the efficacy of CUR alone and turmeric extracts but they were unable to draw definitive conclusions due to the limited number of valid studies (Daily et al., 2016; Shokri-Mashhadi et al., 2021; Wang et al., 2021; Zeng et al., 2021). In the context of CUR’s action on CBR2, Pawar et al. (2022) reported that CUR could selectively act as an agonist on CBR2, reducing inflammation within the myocardium of diabetic mice with myocardial infarction (Pawar et al., 2022). This anti-inflammatory effect was then inhibited by the CBR2 receptor antagonist AM630 (6-Iodopravadoline) (Pawar et al., 2022).

At the time of this publication, no studies have yet explored the role of CBR2 in the observed beneficial properties of CUR or turmeric extracts against OA. This study aimed to investigate the ability of TUR to activate CBR2 and to understand the role of CBR2 in TUR’s anti-inflammatory and antioxidant properties using a CBR2 antagonist in an OA *in vitro* model.

For this study, after conducting a viability assay, 15 ppm TUR was selected as treatment. Based on titration results, this dose corresponded to 2.73 ± 0.39 ppm CUR (7.41 ± 1.06 μM), 0.90 ± 0.13 ppm DMC (2.66 ± 0.38 μM), and 0.79 ± 0.11 ppm BDMC (2.56 ± 0.36 μM). According to the review by Dei Cas and Ghidoni. (2019), CUR and curcuminoids can exhibit a wide range of serum concentrations, ranging from 0.001 to 3.2 ppm, depending on the subject’s health status and administered dosage (Dei Cas and Ghidoni, 2019). The selected TUR dosage falls within this range, ensuring that a relevant concentration has been used.

### 4.2 Interaction with CBR1 and CBR2

In the present study, a docking analysis was conducted on CUR and its analogs targeting CBR2. CUR and curcuminoids showed low predicted binding energies, ranging from −9.6 to −9.7 kcal/mol, suggesting strong potential interactions with CBR2. Typically, predicted binding energies lower than −8.0 kcal/mol indicate possible robust interactions between a ligand and its receptor (Ti et al., 2024). CUR and its analogs showed strong binding affinities for CBR2, similar to the known agonist BCP (Mannino et al., 2023).

The docking analysis also revealed that SR144528, a well-known CBR2 antagonist (Rinaldi-Carmona et al., 1998), interacts strongly with key residues such as TRP258, PHE183, and PHE281, which were highlighted in crystallographic studies by Hua et al. (2020) as crucial for CBR2 conformational changes (Hua et al., 2020). This interaction stabilizes the receptor in its inactive conformation, as reported by Li et al. (2019) (Li et al., 2019), effectively preventing the activation of intracellular Gα inhibitory proteins, which normally inhibit the production of cAMP (Bouaboula et al., 1999). In contrast, CUR and its derivatives (BDMC and DMC) acted as CBR2 agonists, despite being predicted to interact with similar residues within the receptor’s active site as SR144528. Unlike SR144528, these compounds appear to promote the receptor’s active conformation, thereby probably facilitating Gα inhibitory proteins activation. In fact, they maintained low cAMP levels upon FSK stimulation, consistent with the inhibitory effect of CBR2 on adenylyl cyclase (Mallipeddi et al., 2017). This agonistic activity was comparable to that of BCP, another selective CBR2 agonist, which has been well-documented for its therapeutic effects mediated through CBR2 activation (Mannino et al., 2023). Although the docking analysis and cAMP assay suggest strong interactions between TUR components and CBR2, these results should also be validated through other approaches such as X-ray crystallography or site-directed mutagenesis to confirm the binding mechanisms. However, these predicted molecular interactions translated into significant biological effects, particularly in modulating pathways crucial for inflammation and oxidative stress in OA.

### 4.3 Impact on inflammation and oxidative stress

As mentioned before, an important aspect of OA involves the degeneration of cartilage, which can be worsen by oxidative stress. It plays a crucial role in the progression of the disease by promoting chondrocyte apoptosis, as highlighted by Lepetsos and Papavassiliou, 2016. Menadione was used in this study to induce ROS generation, creating an oxidative stress condition (Loor et al., 2010). TUR exhibited a strong antioxidant effect, which was blocked by pretreatment with SR144528. This antioxidant action was likely mediated through HMOX-1 upregulation, as it was shown to be induced by TUR and actively counteracted by the CBR2 antagonist. Since ROS-induced damage, along with pro-inflammatory mechanical stimuli, can trigger pathways like MAPK or NF-κB, it is crucial to explore how TUR modulates these pathways.

The anti-inflammatory effects of TUR, shown by reduced IL-6 and COX-2 mRNA expression during the inflammatory challenge, suggest an interaction with NF-κB signaling. Giacoppo et al. (2017) demonstrated that CBR2 activation in RAW264.7 macrophages reduces oxidative stress by preventing IκB-α phosphorylation, inhibiting the nuclear translocation of NF-κB, and modulating the MAPK pathway (Giacoppo et al., 2017). These effects were reversed by a CBR2 antagonist (Giacoppo et al., 2017). Esposito et al. (2006) previously reported that CBR2 activation reduces p38MAPK phosphorylation, triggering an inhibitory NF-κB signaling mechanism that prevents NF-κB nuclear translocation and subsequently inhibits pro-inflammatory gene transcription (Esposito et al., 2006).

In this study, TUR significantly reduced NFKB1 expression, an effect that was reversed by a CBR2 antagonist. The NFKB1 gene encodes the precursor protein p105, which plays a critical role in NF-κB signaling. Upon activation, p105 undergoes proteolytic processing to generate p50, a subunit that can form various dimers, which are crucial for the transcription of pro-inflammatory genes (Concetti and Wilson, 2018). Additionally, the degradation of p105 not only generates p50 but also releases the associated MAPK kinase TPL-2 (tumor progression locus-2), enabling it to activate the ERK/MAPK cascade, another pathway involved in promoting inflammatory responses (Beinke and Ley, 2004). By reducing the synthesis of p105, TUR may prevent its degradation, thereby blocking TPL-2 and inhibiting the ERK/MAPK cascade.

Mariano et al. (2022) reported that the upregulation and activation of CBR2 are linked to reduced cAMP production, decreased phosphorylated ERK (*p-*ERK), and lower MMP13 levels (Mariano et al., 2022). In OA, increased total ERK levels are associated with chondrocyte hypertrophy and matrix breakdown through MMP upregulation (Wang et al., 2011; Prasadam et al., 2012). Additionally, activation of ERK signaling inhibits apoptosis in chondrocytes, potentially leading to an accumulation of dysfunctional cells (Djouad et al., 2009; Dong et al., 2022). TUR treatment in this study resulted in significant reductions in cAMP, total ERK, and c-Myc protein levels, along with decreased MMP1 and MMP13 mRNA expression, suggesting a role of TUR in the downregulation of total ERK transcription. Li et al. (2017) further indicated that in chondrocytes, the role of *p-*ERK is context-dependent, showing that its activation may not always lead to favorable outcomes in inflammatory responses, thus complicating the interpretation of ERK signaling in osteoarthritis (Li et al., 2017).

These effects were reversed by the CBR2 antagonist, which increased cAMP, total ERK, c-Myc, and MMP expression. This suggests that TUR’s effects on inflammatory markers and MMP are likely mediated by CBR2 activation, emphasizing the key role of CBR2 in the MAPK and NF-κB pathways, as noted in previous studies. Moreover, TUR also significantly upregulated CBR2 mRNA expression, an effect then counteracted by the receptor antagonist. In summary, TUR’s ability to modulate the MAPK and NF-κB pathways via CBR2 activation provides an interesting molecular basis for its anti-inflammatory effects, which could be pivotal in managing OA-related inflammation and cartilage degradation.

Furthermore, Louvet et al. (2011) reported that CBR2 activation can counteract inflammation by upregulating HMOX-1 (Louvet et al., 2011). In this study, TUR also strongly upregulated HMOX-1 mRNA levels, which were reduced by the presence of SR144528. HMOX-1 not only codes for the antioxidant enzyme heme oxygenase 1 (HO-1) but also modulates immune responses (Sebastián et al., 2018), suggesting that its induction by CBR2 activation could be a critical component of the effects observed with TUR (Ryter et al., 2006).

Finally, TUR also reduced the mRNA expression of DAGL-α and MAGL. MAGL is an enzyme involved in converting 2-AG into arachidonic acid. This, coupled with COX-2 downregulation, could lead to reduced prostaglandin production and increased 2-AG accumulation, which may act as an endogenous agonist of CBR2 (Gonsiorek et al., 2000), further enhancing anti-inflammatory effects. However, TUR’s effects on DAGL-α and MAGL appear to be unrelated to CBR2 activation and mRNA upregulation, as pre-treatment with SR144528 did not influence their expression levels during the challenge, compared to TUR alone. Future studies could investigate other signaling pathways or regulatory mechanisms through which TUR may modulate DAGL-α and MAGL expression, providing further insight into its effects on these enzymes.

### 4.4 Challenges, limitations, and future perspectives

Currently, TUR is a promising complementary treatment for OA, with the potential to reduce dependence on nonsteroidal anti-inflammatory drugs and other medications known for their significant side effects, as several clinical studies have been reported on ClinicalTrials.gov. For instance, Paultre et al. (2021) reviewed ten studies comparing turmeric therapy with nonsteroidal anti-inflammatory drugs or no treatment, finding that turmeric significantly improved OA-related pain and joint function from baseline, all while minimizing adverse events (Paultre et al., 2021).

While this study highlights the promising interaction between TUR compounds and CBR2, it is crucial to acknowledge that the role of CBR2 in inflammation is not yet fully understood. While many studies support the anti-inflammatory potential of CBR2 activation, conflicting results in the literature suggest that CBR2’s role in joint inflammation and OA is highly context-dependent. Schuelert et al. (2010) reported that CBR2 agonists unexpectedly exacerbated pain in an OA model, highlighting the complexity of CBR2’s role in pain and inflammation modulation (Schuelert et al., 2010). Moreover, Turcotte et al. (2016) emphasized that CBR2 activation has strong positive effects but they are significantly influenced by the specific cell types and disease models involved (Turcotte et al., 2016). Rzeczycki et al. (2021) observed the upregulation of CBR2 in synovium following joint injury, leading to anti-inflammatory effects (Rzeczycki et al., 2021). On the other hand, Fechtner et al. (2019) found that CBR2 knockdown reduced inflammation in rheumatoid arthritis fibroblasts, indicating that CBR2’s role might differ across inflammatory joint diseases (Fechtner et al., 2019). Mariano et al. (2022) further demonstrated that CBR2 activation in osteoarthritic synoviocytes reduced inflammation through *p-*ERK and MMP13 downregulation, suggesting that CBR2’s signaling pathways may be particularly relevant in OA (Mariano et al., 2022). These varying outcomes underscore the need for further research to delineate the specific conditions under which CBR2 exerts protective versus unfavorable effects. Furthermore, the absence of quantitative protein assessments, such as cytokine measurements via enzyme-linked immunosorbent assay (ELISA) and CBR expression analysis through western blotting, poses a limitation of the present study. These methods could provide a more nuanced understanding of TUR’s impact on specific inflammatory proteins and receptor engagement, thus supporting the observed molecular interactions at a protein level. Future studies incorporating these techniques may yield a clearer picture of TUR’s mechanistic pathways of its anti-inflammatory effects.

It is also important to acknowledge that CUR and curcuminoids exhibit broad-spectrum activity, impacting various intracellular pathways (Deng et al., 2023), which cannot be fully included within the scope of a single *in vitro* study. Deng et al. (2023) demonstrated that CUR influenced more than 1,050 differential mRNAs in transcriptomic analysis during OA in human articular chondrocytes (Deng et al., 2023). Moreover, to enhance the understanding of specific mechanisms, future studies should consider investigating the effects of purified components of TUR on ECS and validating these findings in appropriate *in vivo* models.

Another critical challenge in translating *in vitro* findings into clinical practice is the bioavailability of CUR and its analogs. Despite their *in vitro* effects, the poor systemic bioavailability of these compounds poses a significant challenge to their efficacy *in vivo*. Various strategies, including the development of nanoparticles, liposomes, and phospholipid complexes, have been explored to enhance the bioavailability of TUR components (Bučević Popović et al., 2024). However, concerns have been raised about formulations that enhance CUR’s bioavailability, potentially leading to an increased risk of adverse effects (Turck et al., 2021).

In conclusion, while this study offers promising insights into TUR’s potential mechanisms, it also underscores the complexity of CBR2’s role in inflammation, highlighting the need for ongoing research to fully understand it.

## 5 Conclusion

This study provides evidence that turmeric exhibits anti-inflammatory and antioxidant properties through the activation of CBR2. The docking analysis confirmed strong interactions between CUR and its analogs with CBR2, aligning with the observed biological effects. TUR’s ability to upregulate HMOX-1, downregulate MMP1 and MMP13, and reduce NFKB1, IL-6, and COX-2 mRNA expression, all of which were reversed by a CBR2 antagonist, underscores the crucial role of CBR2 in mediating these effects. However, the translation of these *in vitro* results into clinical practice will require comprehensive *in vivo* and clinical studies and careful consideration of pharmacokinetic challenges. Addressing these factors is critical to exploit the potential of TUR in OA management.

## Data Availability

The raw data supporting the conclusions of this article will be made available by the authors, without undue reservation.

## Glossary

BCP: β-Caryophyllene
BDMC: Bisdemethoxycurcumin
cAMP: Cyclic Adenosine Monophosphate
CBR: Cannabinoid Receptor
COX-2: Cyclooxygenase-2
CUR: Curcumin
DAPI: 4′,6-diamidino-2-phenylindole
DAGL-α: Diacylglycerol Lipase Alpha
DMC: Demethoxycurcumin
DMSO: Dimethyl sulfoxide
DPBS: Dulbecco’s Phosphate-Buffered Saline
ECS: Endocannabinoid System
ERK: Extracellular Signal-Regulated Kinase
FSK: Forskolin
HMOX-1: Heme Oxygenase −1
IL: Interleukin
IBMX: 3-isobutyl-1-methylxanthine
MAGL: Monoacylglycerol Lipase
MAPK: Mitogen-Activated Protein Kinase
MMP: Matrix Metalloprotease
NF-κB: Nuclear Factor kappa-light-chain-enhancer of activated B cells
NFKB1: Nuclear Factor kappa-light-chain-enhancer of activated B cells subunit 1
NRF2: Nuclear Factor Erythroid 2–Related Factor 2
OA: Osteoarthritis
ROS: Reactive Oxygen Species
RPL13: Ribosomal Protein L13
qPCR: Quantitative Polymerase Chain Reaction
PPAR-γ: Peroxisome Proliferator-Activated Receptor Gamma
SEM: Standard Error of the Mean
SW-1353: Human Chondrosarcoma Cell Line
TBP: TATA-binding protein
TNF-α: Tumor Necrosis Factor-alpha
TUR: Turmeric Oleoresin
