# Supplementary material for: Activation of cannabinoid receptor 2 by turmeric oleoresin reduces inflammation and oxidative stress in an osteoarthritis in vitro model
Source: Front Pharmacol. 2024 Dec 9;15:1488254. doi: 10.3389/fphar.2024.1488254 (PMC11664362; doi:10.3389/fphar.2024.1488254)
Supplement: Supplementary file 1 [file DataSheet1.docx]

Supplementary Material

# Supplementary Figures and Tables

## Supplementary Figures

**Supplementary Figure 1. Oxidative stress challenge optimization.** Menadione 100 μM was chosen as the oxidative stress induction treatment due to its ability to induce the highest significant oxidative stress response.


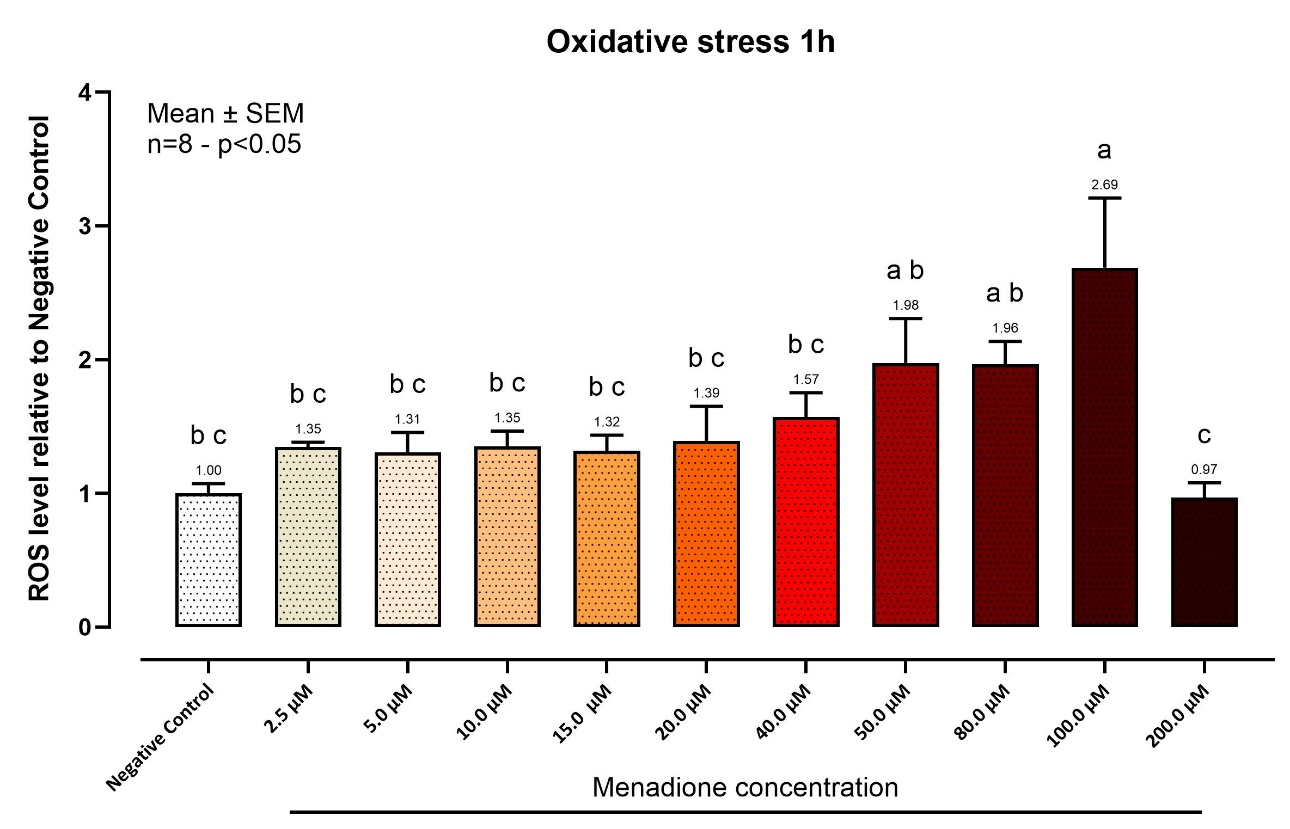


ROS levels were assessed using CellROX^®^ Deep Red Reagent (Thermo Fisher Scientific, Milan, Italy) according to the manufacturer's guidelines. Briefly, CellROX^®^ Deep Red Reagent is a cell-permeable, non-fluorescent reagent that becomes fluorescent upon oxidation by ROS, with an emission maximum of around 665 nm. SW-1353 cells were seeded in 96-well plates at a density of 1 × 10^4^ cells/well and subjected to a challenge 48 hours post-seeding. Then, menadione at increasing concentrations was introduced as a ROS-inducing agent for 1 hour. Subsequently, the medium containing menadione was replaced with fresh media containing 5 μM CellROX^®^ Deep Red Reagent, and the cells were incubated for 30 minutes at 37 °C. The medium was then removed, and the cells were washed three times with Dulbecco’s phosphate-buffered saline (DPBS). Fluorescence values were recorded using a Varioskan LUX (Thermo Fisher Scientific, Milan, Italy).

Data exhibited a normal distribution. They are presented as mean ± SEM (n=8) and represent the ROS level relative to the Negative Control. Statistical analysis was performed using one-way ANOVA followed by Tukey’s multiple comparisons test (p<0.05). Different letters indicate significant differences.

**Supplementary Figure 2. Inflammatory Challenge optimization.** A combination of IL-1β and TNF-α, both at 10 ng/mL, was chosen as the pro-inflammatory treatment due to its ability to induce a potent inflammatory response. Moreover, these cytokines are considered primary mediators of inflammation in OA.


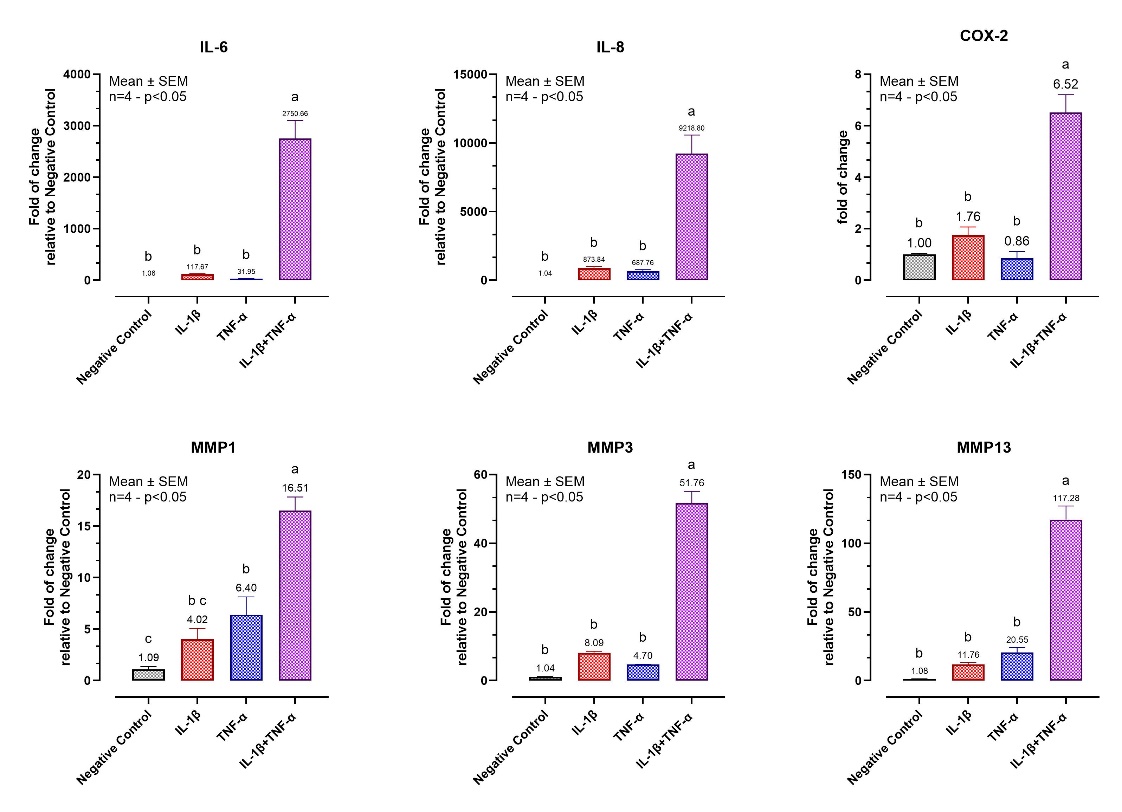


Cells were seeded into 24-well plates at a density of 5 × 10^4^ cells/well and subjected to a challenge 48 hours post-seeding. The cells were categorized into four distinct groups: (1) Negative Control, (2) IL-1β 10 ng/mL, (3) TNF-α 10 ng/mL, and (4) IL-1β+TNF-α both at 10 ng/mL. After 24 hours, cells were rinsed with DPBS, harvested for RNA extraction, and subjected to qPCR analysis. Data are presented as mean ± SEM (n=4) and represent the fold change relative to the Negative Control. Statistical analysis was performed using one-way ANOVA followed by Tukey’s multiple comparisons test (p<0.05). Different letters indicate significant differences. IL = Interleukin; COX-2 = Cyclooxygenase-2; MMP = Matrix metalloproteinase.

## Supplementary Tables

**Supplementary Table 1. Docking analysis on CBR1.** The docking analysis of bisdemethoxycurcumin (BDMC), demethoxycurcumin (DMC), and curcumin (CUR) against the Cannabinoid Receptor 1 (CBR1), in comparison with rimonabant, provides valuable insights into their observed failure to activate CBR1 in cAMP assays. These compounds exhibit slightly less favorable binding energies than rimonabant, ranging from -9.2 to -9.5 kcal/mol compared to rimonabant's -11.6 kcal/mol, yet they interact with several key residues critical for maintaining the receptor in its inactive state. Notably, all four compounds engage with residues such as PHE102, MET103, PHE268, and TRP356, which are part of the structural network that stabilizes the inactive conformation of CBR1.

BDMC, DMC, and CUR, like rimonabant, also interact with residues SER383, CYS386, and LEU387, which contribute to locking the receptor in an inactive state. While rimonabant shows additional interactions with residues like SER123 and THR197, which may enhance its binding affinity and inhibitory effect, BDMC, DMC, and CUR's interactions with the aforementioned critical residues are still sufficient to prevent the receptor from transitioning to an active state.

In contrast, β-caryophyllene, which is known as a CBR2-selective agonist, exhibits a lower binding energy of -8.0 kcal/mol when docked to CBR1. BCP interacts with several of the same residues, such as PHE102, PHE268, and TRP356, but these interactions appear insufficient to stabilize CBR1 in either an active or inactive conformation, possibly due to the lack of engagement with other key residues like SER123 or THR197.

| Compound | Binding Energy (kcal/mol) | Contact residues |
| --- | --- | --- |
| Rimonabant | -11.6 | PHE102, MET103, ILE105, ILE119, SER123, GLY166, SER167, ILE169, PHE170, SER173, PHE174, HIS178, LEU193, VAL196, THR197, PHE268, TRP356, LEU359, PHE379, ALA380, SER383, MET384, CYS386, LEU387, SER390 |
| Bisdemethoxycurcumin | -9.5 | ASN101, PHE102, MET103, ASP104, ILE105, ASP163, GLY166, SER167, PHE170, PHE189, LEU193, VAL196, ILE267, PHE268, PRO269, TRP356, PHE379, SER383, CYS386, LEU387, SER390 |
| Demethoxycurcumin | -9.4 | ASN101, PHE102, MET103, ASP104, ILE105, GLU106, ASP163, GLY166, SER167, PHE170, PHE189, VAL196, ILE267, PHE268, PRO269, TRP356, LEU359, PHE379, SER383, CYS386, LEU387, SER390 |
| Curcumin | -9.2 | ASN101, PHE102, MET103, ASP104, ILE105, GLU106, PHE189, LEU193, VAL196, THR197, ILE267, PHE268, PRO269, ILE271, TYR275, LEU276, TRP279, TRP356, LEU359, MET363, PHE379, SER383 |
| β-Caryophyllene | -8.0 | PHE102, MET103, GLY166, PHE170, LEU193, VAL196, PHE268, TRP356, LEU359, PHE379, SER383, CYS386, LEU387 |

Binding energies and contact residues computed with CB-Dock2 on Cannabinoid receptor 1 (PDB ID: 5TGZ). Rimonabant = CBR1 Antagonist. Important residues serving as “switches” for receptor inactivation are underlined. Molecular docking has been performed with CB-Dock2 online tool (https://cadd.labshare.cn/cb-dock2/index.php) through the “Structure-based Blind Docking” function. The CBR1 (PDB ID: 5TGZ) protein structure was downloaded from the PDB database (https://www.rcsb.org/) and the 3D molecular structures of BCP, Rimonabant, CUR, DMC, and BDMC from PubChem (<https://pubchem.ncbi.nlm.nih.gov/>).
